# Supplementary material for: Kinetic Studies of Antioxidant Properties of Ovothiol A
Source: Antioxidants (Basel). 2021 Sep 15;10(9):1470. doi: 10.3390/antiox10091470 (PMC8470380; doi:10.3390/antiox10091470)
Supplement: Supplementary file 1 [file antioxidants-10-01470-s001.zip › antioxidants-1366924-supplementary.pdf]

Supplementary Information

for

Kinetic studies of antioxidant properties of ovothiol A

Nataliya A. Osik,[1,2] Ekaterina A. Zelentsova,[1,2] Yuri P. Tsentalovich[1,2,*]

[1]International Tomography Center SB RAS, Institutskaya 3a, Novosibirsk 630090, Russia

[2]Novosibirsk State University, Pirogova 2, Novosibirsk 630090, Russia

* Corresponding author, e-mail: yura@tomo.nsc.ru

Table of content

Supplementary Figure S1. Absorption spectra of aqueous solution (pH 7.4, T = 15ºC) containing 3.7×10^{-5} M OSSO before addition of GSH, and 50 s and 189 s after the addition of 0.2 mM GSH.

Supplementary Figure S2. Dependence of the pseudo-first order rate constant k_1' on GSH concentration.

Supplementary Figure S3. Dependence of the pseudo-first order rate constant k_2' on GSH concentration.

Supplementary Figure S4. Absorption spectra of aqueous solution (pH 7.4, T = 25 ºC) containing 4.6×10^{-5} M OSH and 2.3×10^{-5} M GSSG before addition of H_2O_2, and 156 s and 258 s after the addition of 6 mM H_2O_2.

Supplementary Figure S5. Dependence of the pseudo-first order rate constant k_3' on H_2O_2 concentration.

Supplementary Scheme S1. Structures of compounds used or detected in this work: oxidized ovothiol (OSSO), reduced glutathione (GSH), sulfenic acid (OSOH), and ovothiol-glutathione adduct (OSSG).

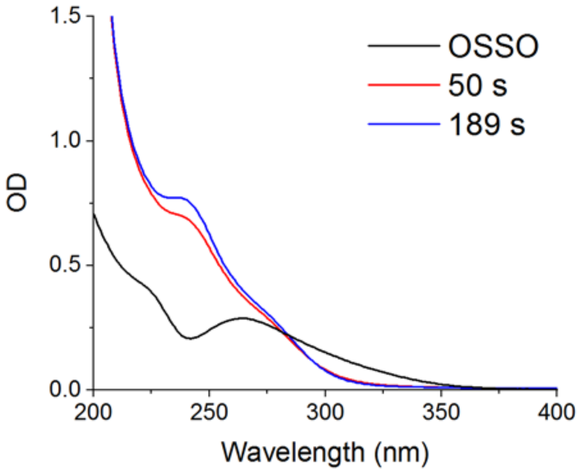

Figure S1. Absorption spectra of aqueous solution (pH 7.4, T = 15ºC) containing 3.7×10^{-5} M OSSO before addition of GSH, and 50 s and 189 s after the addition of 0.2 mM GSH.

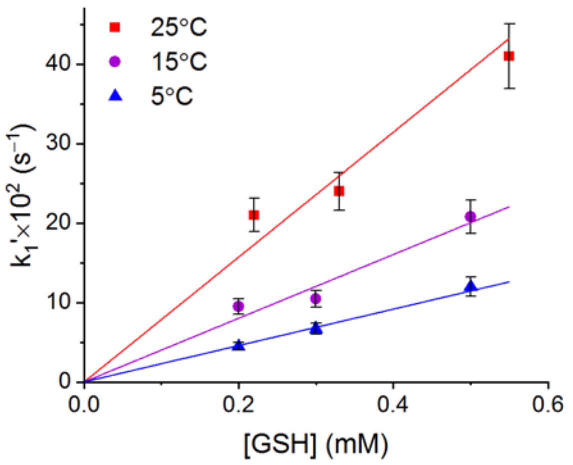

Figure S2. Dependence of the pseudo-first order rate constant k_1' on GSH concentration.

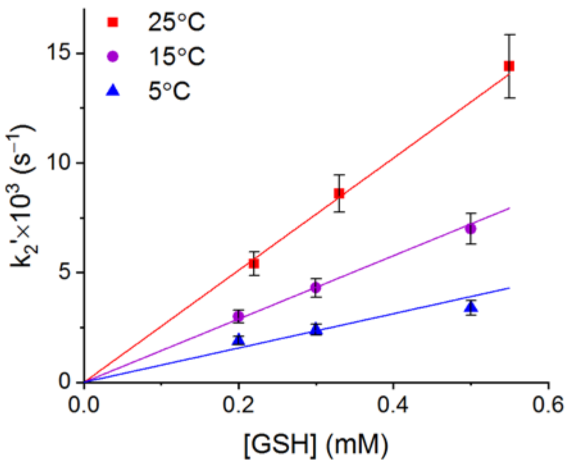

Figure S3. Dependence of the pseudo-first order rate constant k_2' on GSH concentration.

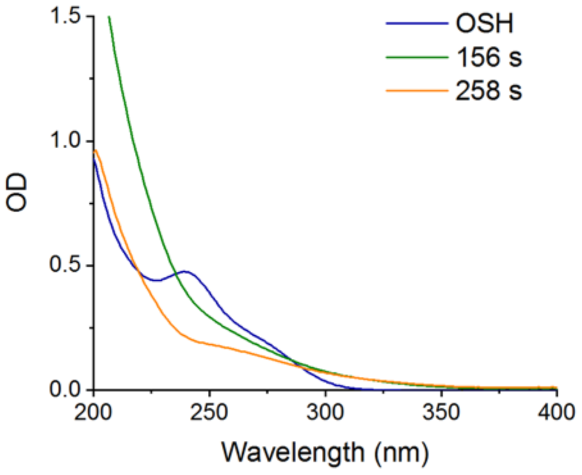

Figure S4. Absorption spectra of aqueous solution (pH 7.4, T = 25 ºC) containing 4.6×10^{-5} M OSH and 2.3×10^{-5} M GSSG before addition of H_2O_2, and 156 s and 258 s after the addition of 6 mM H_2O_2.

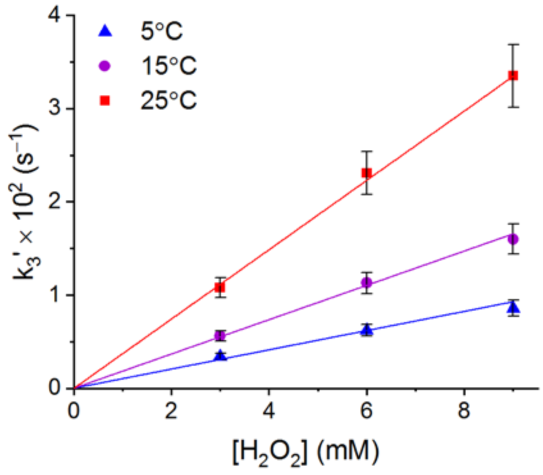

Figure S5. Dependence of the pseudo-first order rate constant k_3' on H_2O_2 concentration.

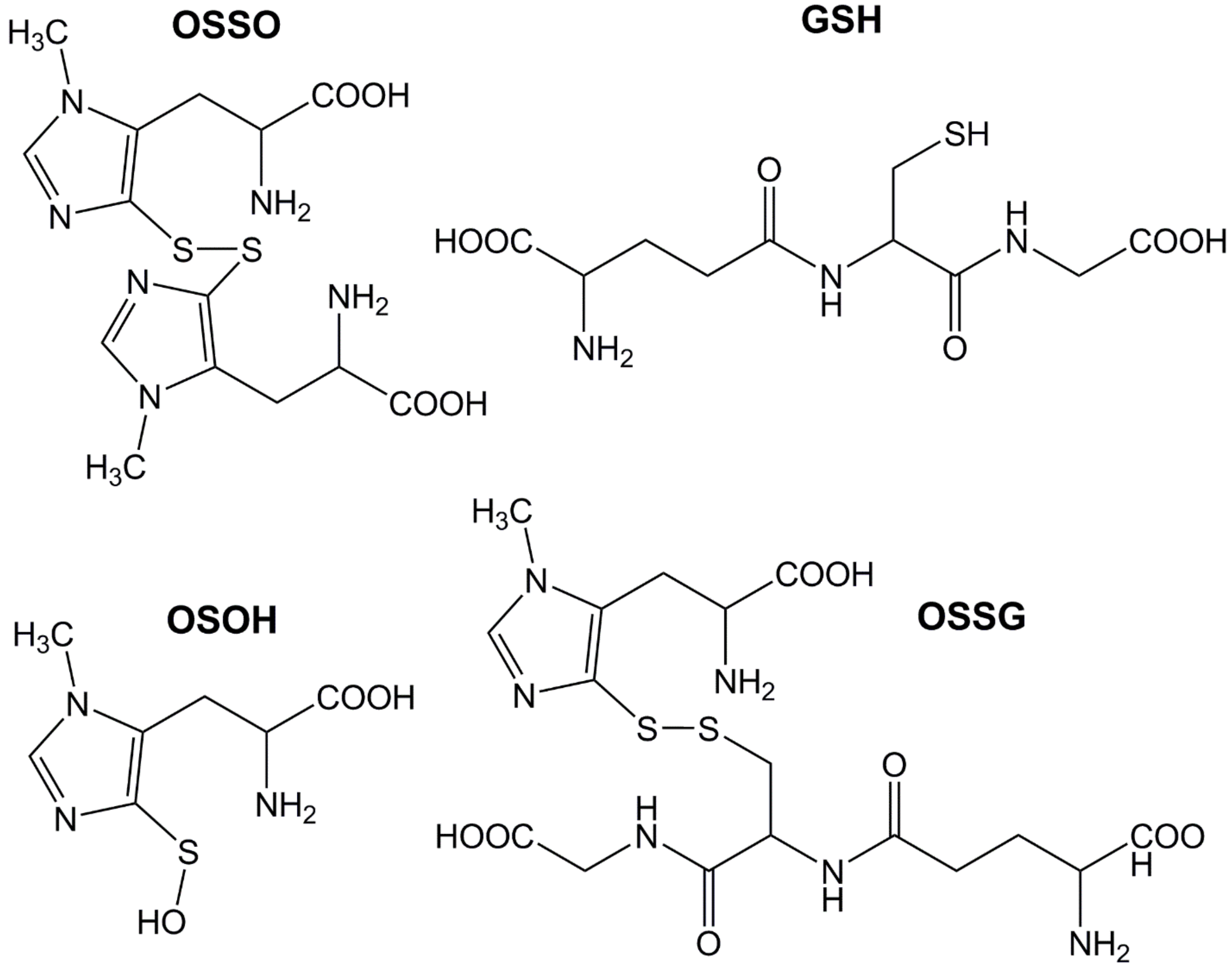

Scheme S1. Structures of compounds used or detected in this work: oxidized ovothiol (OSSO), reduced glutathione (GSH), sulfenic acid (OSOH), and ovothiol-glutathione adduct (OSSG).
